# Supplementary material for: Long COVID risk by pre-infection symptoms and functional status: A retrospective cohort study of data from the All of Us Research Program
Source: PLoS One. 2026 Jun 16;21(6):e0330793. doi: 10.1371/journal.pone.0330793 (PMC13271467; doi:10.1371/journal.pone.0330793)
Supplement: S2 Table — All laboratory observations, COVID-19 Participant Experience (COPE) survey item responses, and diagnostic codes in SNOMED vocabulary indicating either SARS-CoV-2 infection or COVID-19 illness. Generated using queries for lab values, survey item responses, and diagnostic code incidences via the All of Us Researcher Workbench dataset builder. (DOCX) [file pone.0330793.s002.docx]

**Table A.1. Identification of cohort - COVID-19 illness indicators**

| **Name** | **Concept ID** | **Vocab** | **Code** | **Roll-up Count** | **Item Count** |
| --- | --- | --- | --- | --- | --- |
| **Laboratory observations** |  |  |  |  |  |
| 2019-ncov coronavirus, sars-cov-2/2019-ncov (covid-19), any technique, multiple types or subtypes (includes all targets), non-cdc | [40218804](https://databrowser.researchallofus.org/ehr/labs-and-measurements/40218804) | HCPCS | U0002 | 0 | 1,020 |
| 2019-nCoV Coronavirus, SARS-CoV-2/2019-nCoV (COVID-19), any technique, multiple types or subtypes (includes all targets), non-CDC, making use of high throughput technologies as described by CMS-2020-01-R | [704058](https://databrowser.researchallofus.org/ehr/labs-and-measurements/704058) | HCPCS | U0004 | 0 | 1 |
| Infectious agent detection by nucleic acid (DNA or RNA); severe acute respiratory syndrome coronavirus 2 (SARS-CoV-2) (Coronavirus disease [COVID-19]), amplified probe technique, making use of high throughput technologies as described by CMS-2020-01-R | [704059](https://databrowser.researchallofus.org/ehr/labs-and-measurements/704059) | HCPCS | U0003 | 0 | 1,143 |
| Influenza virus A and B and SARS-CoV-2 (COVID-19) and SARS-related CoV RNA panel | [36660845](https://databrowser.researchallofus.org/ehr/labs-and-measurements/36660845) | LOINC | LP418968-6 | 985 | 0 |
| Influenza virus A and B and SARS-CoV-2 (COVID-19) and SARS-related CoV RNA panel - Respiratory specimen by NAA with probe detection | [36661384](https://databrowser.researchallofus.org/ehr/labs-and-measurements/36661384) | LOINC | 95380-2 | 0 | 985 |
| Influenza virus A and B and SARS-CoV-2 (COVID-19) and SARS-related CoV RNA panel \| Respiratory specimen \| Microbiology Panels | [36661218](https://databrowser.researchallofus.org/ehr/labs-and-measurements/36661218) | LOINC | LP419290-4 | 985 | 0 |
| Influenza virus A and B and SARS-CoV-2 (COVID-19) RNA panel - Respiratory specimen by NAA with probe detection | [36661376](https://databrowser.researchallofus.org/ehr/labs-and-measurements/36661376) | LOINC | 95422-2 | 0 | 330 |
| Measurement of Severe acute respiratory syndrome coronavirus 2 (SARS-CoV-2) | [756055](https://databrowser.researchallofus.org/ehr/labs-and-measurements/756055) | OMOP Extension | OMOP4873969 | 0 | 384 |
| Measurement of Severe acute respiratory syndrome coronavirus 2 antibody | [37310258](https://databrowser.researchallofus.org/ehr/labs-and-measurements/37310258) | SNOMED | 1.24046E+15 | 0 | 263 |
| SARS-CoV-2 (COVID-19) | [36662140](https://databrowser.researchallofus.org/ehr/labs-and-measurements/36662140) | LOINC | LP417540-4 | 92,959 | 0 |
| SARS-CoV-2 (COVID-19) Ab | [36661733](https://databrowser.researchallofus.org/ehr/labs-and-measurements/36661733) | LOINC | LP417914-1 | 9,362 | 0 |
| SARS-CoV-2 (COVID-19) Ab [Interpretation] in Serum or Plasma | [723480](https://databrowser.researchallofus.org/ehr/labs-and-measurements/723480) | LOINC | 94661-6 | 0 | 3,037 |
| SARS-CoV-2 (COVID-19) Ab [Presence] in Serum or Plasma by Immunoassay | [586515](https://databrowser.researchallofus.org/ehr/labs-and-measurements/586515) | LOINC | 94762-2 | 0 | 1,146 |
| SARS-CoV-2 (COVID-19) Ab [Units/volume] in Serum or Plasma by Immunoassay | [586522](https://databrowser.researchallofus.org/ehr/labs-and-measurements/586522) | LOINC | 94769-7 | 0 | 309 |
| SARS-CoV-2 (COVID-19) Ab \| Serum or Plasma \| Microbiology | [36661221](https://databrowser.researchallofus.org/ehr/labs-and-measurements/36661221) | LOINC | LP418684-9 | 4,130 | 0 |
| SARS-CoV-2 (COVID-19) Ab panel | [36661883](https://databrowser.researchallofus.org/ehr/labs-and-measurements/36661883) | LOINC | LP418122-0 | 2 | 0 |
| SARS-CoV-2 (COVID-19) Ab panel - Serum or Plasma by Immunoassay | [706179](https://databrowser.researchallofus.org/ehr/labs-and-measurements/706179) | LOINC | 94504-8 | 0 | 2 |
| SARS-CoV-2 (COVID-19) Ab panel \| Serum or Plasma \| Microbiology Panels | [36661105](https://databrowser.researchallofus.org/ehr/labs-and-measurements/36661105) | LOINC | LP419286-2 | 2 | 0 |
| SARS-CoV-2 (COVID-19) Ag | [36661764](https://databrowser.researchallofus.org/ehr/labs-and-measurements/36661764) | LOINC | LP418019-8 | 1,262 | 0 |
| SARS-CoV-2 (COVID-19) Ag [Presence] in Respiratory specimen by Rapid immunoassay | [723477](https://databrowser.researchallofus.org/ehr/labs-and-measurements/723477) | LOINC | 94558-4 | 0 | 1,222 |
| SARS-CoV-2 (COVID-19) Ag [Presence] in Upper respiratory specimen by Immunoassay | [36032419](https://databrowser.researchallofus.org/ehr/labs-and-measurements/36032419) | LOINC | 96119-3 | 0 | 40 |
| SARS-CoV-2 (COVID-19) Ag \| Respiratory specimen \| Microbiology | [36660801](https://databrowser.researchallofus.org/ehr/labs-and-measurements/36660801) | LOINC | LP418693-0 | 1,222 | 0 |
| SARS-CoV-2 (COVID-19) Ag \| Upper respiratory specimen \| Microbiology | [36033457](https://databrowser.researchallofus.org/ehr/labs-and-measurements/36033457) | LOINC | LP420931-0 | 40 | 0 |
| SARS-CoV-2 (COVID-19) clade | [1620066](https://databrowser.researchallofus.org/ehr/labs-and-measurements/1620066) | LOINC | LP422736-1 | 47 | 0 |
| SARS-CoV-2 (COVID-19) clade [Type] in Specimen by Molecular genetics method | [36033653](https://databrowser.researchallofus.org/ehr/labs-and-measurements/36033653) | LOINC | 96896-6 | 0 | 47 |
| SARS-CoV-2 (COVID-19) clade \| XXX \| Microbiology | [1618285](https://databrowser.researchallofus.org/ehr/labs-and-measurements/1618285) | LOINC | LP427406-6 | 47 | 0 |
| SARS-CoV-2 (COVID-19) IgA | [36662109](https://databrowser.researchallofus.org/ehr/labs-and-measurements/36662109) | LOINC | LP418430-7 | 167 | 0 |
| SARS-CoV-2 (COVID-19) IgA \| Serum or Plasma \| Microbiology | [36660931](https://databrowser.researchallofus.org/ehr/labs-and-measurements/36660931) | LOINC | LP418685-6 | 167 | 0 |
| SARS-CoV-2 (COVID-19) IgA Ab [Presence] in Serum or Plasma by Immunoassay | [723473](https://databrowser.researchallofus.org/ehr/labs-and-measurements/723473) | LOINC | 94562-6 | 0 | 167 |
| SARS-CoV-2 (COVID-19) IgG | [36661886](https://databrowser.researchallofus.org/ehr/labs-and-measurements/36661886) | LOINC | LP417915-8 | 5,025 | 0 |
| SARS-CoV-2 (COVID-19) IgG \| Serum or Plasma \| Microbiology | [36661046](https://databrowser.researchallofus.org/ehr/labs-and-measurements/36661046) | LOINC | LP418688-0 | 3,500 | 0 |
| SARS-CoV-2 (COVID-19) IgG \| Serum, Plasma or Blood \| Microbiology | [36660768](https://databrowser.researchallofus.org/ehr/labs-and-measurements/36660768) | LOINC | LP418689-8 | 2,090 | 0 |
| SARS-CoV-2 (COVID-19) IgG Ab [Presence] in Serum or Plasma by Immunoassay | [723474](https://databrowser.researchallofus.org/ehr/labs-and-measurements/723474) | LOINC | 94563-4 | 0 | 2,920 |
| SARS-CoV-2 (COVID-19) IgG Ab [Presence] in Serum, Plasma or Blood by Rapid immunoassay | [706181](https://databrowser.researchallofus.org/ehr/labs-and-measurements/706181) | LOINC | 94507-1 | 0 | 2,090 |
| SARS-CoV-2 (COVID-19) IgG Ab [Units/volume] in Serum or Plasma by Immunoassay | [706177](https://databrowser.researchallofus.org/ehr/labs-and-measurements/706177) | LOINC | 94505-5 | 0 | 749 |
| SARS-CoV-2 (COVID-19) IgG+IgM | [36661646](https://databrowser.researchallofus.org/ehr/labs-and-measurements/36661646) | LOINC | LP417956-2 | 991 | 0 |
| SARS-CoV-2 (COVID-19) IgG+IgM \| Serum or Plasma \| Microbiology | [36660914](https://databrowser.researchallofus.org/ehr/labs-and-measurements/36660914) | LOINC | LP418690-6 | 991 | 0 |
| SARS-CoV-2 (COVID-19) IgG+IgM Ab [Presence] in Serum or Plasma by Immunoassay | [723479](https://databrowser.researchallofus.org/ehr/labs-and-measurements/723479) | LOINC | 94547-7 | 0 | 991 |
| SARS-CoV-2 (COVID-19) IgM | [36661975](https://databrowser.researchallofus.org/ehr/labs-and-measurements/36661975) | LOINC | LP417916-6 | 396 | 0 |
| SARS-CoV-2 (COVID-19) IgM \| Serum or Plasma \| Microbiology | [36661274](https://databrowser.researchallofus.org/ehr/labs-and-measurements/36661274) | LOINC | LP418691-4 | 299 | 0 |
| SARS-CoV-2 (COVID-19) IgM \| Serum, Plasma or Blood \| Microbiology | [36660777](https://databrowser.researchallofus.org/ehr/labs-and-measurements/36660777) | LOINC | LP418692-2 | 97 | 0 |
| SARS-CoV-2 (COVID-19) IgM Ab [Presence] in Serum or Plasma by Immunoassay | [723475](https://databrowser.researchallofus.org/ehr/labs-and-measurements/723475) | LOINC | 94564-2 | 0 | 275 |
| SARS-CoV-2 (COVID-19) IgM Ab [Presence] in Serum, Plasma or Blood by Rapid immunoassay | [706180](https://databrowser.researchallofus.org/ehr/labs-and-measurements/706180) | LOINC | 94508-9 | 0 | 97 |
| SARS-CoV-2 (COVID-19) IgM Ab [Units/volume] in Serum or Plasma by Immunoassay | [706178](https://databrowser.researchallofus.org/ehr/labs-and-measurements/706178) | LOINC | 94506-3 | 0 | 37 |
| SARS-CoV-2 (COVID-19) lineage | [1619966](https://databrowser.researchallofus.org/ehr/labs-and-measurements/1619966) | LOINC | LP422739-5 | 17 | 0 |
| SARS-CoV-2 (COVID-19) lineage [Identifier] in Specimen by Molecular genetics method | [36033652](https://databrowser.researchallofus.org/ehr/labs-and-measurements/36033652) | LOINC | 96895-8 | 0 | 17 |
| SARS-CoV-2 (COVID-19) lineage \| XXX \| Microbiology | [1618914](https://databrowser.researchallofus.org/ehr/labs-and-measurements/1618914) | LOINC | LP427405-8 | 17 | 0 |
| SARS-CoV-2 (COVID-19) N gene | [36661396](https://databrowser.researchallofus.org/ehr/labs-and-measurements/36661396) | LOINC | LP417599-0 | 1,151 | 0 |
| SARS-CoV-2 (COVID-19) N gene [Presence] in Nasopharynx by NAA with probe detection | [715272](https://databrowser.researchallofus.org/ehr/labs-and-measurements/715272) | LOINC | 94760-6 | 0 | 36 |
| SARS-CoV-2 (COVID-19) N gene [Presence] in Nose by NAA with probe detection | [757678](https://databrowser.researchallofus.org/ehr/labs-and-measurements/757678) | LOINC | 95409-9 | 0 | 9 |
| SARS-CoV-2 (COVID-19) N gene [Presence] in Respiratory specimen by NAA with probe detection | [706161](https://databrowser.researchallofus.org/ehr/labs-and-measurements/706161) | LOINC | 94533-7 | 0 | 671 |
| SARS-CoV-2 (COVID-19) N gene [Presence] in Respiratory specimen by Nucleic acid amplification using CDC primer-probe set N1 | [586524](https://databrowser.researchallofus.org/ehr/labs-and-measurements/586524) | LOINC | 94756-4 | 0 | 468 |
| SARS-CoV-2 (COVID-19) N gene \| Nasopharynx \| Microbiology | [36660752](https://databrowser.researchallofus.org/ehr/labs-and-measurements/36660752) | LOINC | LP418702-9 | 36 | 0 |
| SARS-CoV-2 (COVID-19) N gene \| Nose \| Microbiology | [36660970](https://databrowser.researchallofus.org/ehr/labs-and-measurements/36660970) | LOINC | LP419179-9 | 9 | 0 |
| SARS-CoV-2 (COVID-19) N gene \| Respiratory specimen \| Microbiology | [36661286](https://databrowser.researchallofus.org/ehr/labs-and-measurements/36661286) | LOINC | LP418703-7 | 1,107 | 0 |
| SARS-CoV-2 (COVID-19) ORF1ab region | [36661401](https://databrowser.researchallofus.org/ehr/labs-and-measurements/36661401) | LOINC | LP417906-7 | 1,267 | 0 |
| SARS-CoV-2 (COVID-19) ORF1ab region [Presence] in Respiratory specimen by NAA with probe detection | [723478](https://databrowser.researchallofus.org/ehr/labs-and-measurements/723478) | LOINC | 94559-2 | 0 | 1,265 |
| SARS-CoV-2 (COVID-19) ORF1ab region [Presence] in Specimen by NAA with probe detection | [723464](https://databrowser.researchallofus.org/ehr/labs-and-measurements/723464) | LOINC | 94639-2 | 0 | 2 |
| SARS-CoV-2 (COVID-19) ORF1ab region \| Respiratory specimen \| Microbiology | [36661250](https://databrowser.researchallofus.org/ehr/labs-and-measurements/36661250) | LOINC | LP418706-0 | 1,265 | 0 |
| SARS-CoV-2 (COVID-19) ORF1ab region \| XXX \| Microbiology | [36661194](https://databrowser.researchallofus.org/ehr/labs-and-measurements/36661194) | LOINC | LP418707-8 | 2 | 0 |
| SARS-CoV-2 (COVID-19) RdRp gene | [36661801](https://databrowser.researchallofus.org/ehr/labs-and-measurements/36661801) | LOINC | LP417598-2 | 3,774 | 0 |
| SARS-CoV-2 (COVID-19) RdRp gene [Presence] in Respiratory specimen by NAA with probe detection | [706160](https://databrowser.researchallofus.org/ehr/labs-and-measurements/706160) | LOINC | 94534-5 | 0 | 3,764 |
| SARS-CoV-2 (COVID-19) RdRp gene [Presence] in Specimen by NAA with probe detection | [706173](https://databrowser.researchallofus.org/ehr/labs-and-measurements/706173) | LOINC | 94314-2 | 0 | 18 |
| SARS-CoV-2 (COVID-19) RdRp gene \| Respiratory specimen \| Microbiology | [36660902](https://databrowser.researchallofus.org/ehr/labs-and-measurements/36660902) | LOINC | LP418708-6 | 3,764 | 0 |
| SARS-CoV-2 (COVID-19) RdRp gene \| XXX \| Microbiology | [36660887](https://databrowser.researchallofus.org/ehr/labs-and-measurements/36660887) | LOINC | LP418709-4 | 18 | 0 |
| SARS-CoV-2 (COVID-19) RNA | [36661507](https://databrowser.researchallofus.org/ehr/labs-and-measurements/36661507) | LOINC | LP417541-2 | 89,445 | 0 |
| SARS-CoV-2 (COVID-19) RNA [Cycle Threshold #] in Respiratory specimen by NAA with probe detection | [586528](https://databrowser.researchallofus.org/ehr/labs-and-measurements/586528) | LOINC | 94745-7 | 0 | 1 |
| SARS-CoV-2 (COVID-19) RNA [Presence] in Nasopharynx by NAA with non-probe detection | [723476](https://databrowser.researchallofus.org/ehr/labs-and-measurements/723476) | LOINC | 94565-9 | 0 | 2,005 |
| SARS-CoV-2 (COVID-19) RNA [Presence] in Nasopharynx by NAA with probe detection | [586526](https://databrowser.researchallofus.org/ehr/labs-and-measurements/586526) | LOINC | 94759-8 | 0 | 273 |
| SARS-CoV-2 (COVID-19) RNA [Presence] in Respiratory specimen by NAA with probe detection | [706163](https://databrowser.researchallofus.org/ehr/labs-and-measurements/706163) | LOINC | 94500-6 | 0 | 63,160 |
| SARS-CoV-2 (COVID-19) RNA [Presence] in Saliva (oral fluid) by Sequencing | [715261](https://databrowser.researchallofus.org/ehr/labs-and-measurements/715261) | LOINC | 94822-4 | 0 | 107 |
| SARS-CoV-2 (COVID-19) RNA [Presence] in Specimen by NAA with probe detection | [706170](https://databrowser.researchallofus.org/ehr/labs-and-measurements/706170) | LOINC | 94309-2 | 0 | 27,394 |
| SARS-CoV-2 (COVID-19) RNA \| Nasopharynx \| Microbiology | [36661317](https://databrowser.researchallofus.org/ehr/labs-and-measurements/36661317) | LOINC | LP418694-8 | 2,274 | 0 |
| SARS-CoV-2 (COVID-19) RNA \| Respiratory specimen \| Microbiology | [36661115](https://databrowser.researchallofus.org/ehr/labs-and-measurements/36661115) | LOINC | LP418695-5 | 63,160 | 0 |
| SARS-CoV-2 (COVID-19) RNA \| Saliva \| Microbiology | [36660966](https://databrowser.researchallofus.org/ehr/labs-and-measurements/36660966) | LOINC | LP418696-3 | 107 | 0 |
| SARS-CoV-2 (COVID-19) RNA \| XXX \| Microbiology | [36661244](https://databrowser.researchallofus.org/ehr/labs-and-measurements/36661244) | LOINC | LP418698-9 | 27,394 | 0 |
| SARS-CoV-2 (COVID-19) RNA panel | [36661522](https://databrowser.researchallofus.org/ehr/labs-and-measurements/36661522) | LOINC | LP417539-6 | 5,080 | 0 |
| SARS-CoV-2 (COVID-19) RNA panel - Respiratory specimen by NAA with probe detection | [706158](https://databrowser.researchallofus.org/ehr/labs-and-measurements/706158) | LOINC | 94531-1 | 0 | 1,342 |
| SARS-CoV-2 (COVID-19) RNA panel - Specimen by NAA with probe detection | [706169](https://databrowser.researchallofus.org/ehr/labs-and-measurements/706169) | LOINC | 94306-8 | 0 | 3,740 |
| SARS-CoV-2 (COVID-19) RNA panel \| Respiratory specimen \| Microbiology Panels | [36661036](https://databrowser.researchallofus.org/ehr/labs-and-measurements/36661036) | LOINC | LP419288-8 | 1,342 | 0 |
| SARS-CoV-2 (COVID-19) RNA panel \| XXX \| Microbiology Panels | [36660924](https://databrowser.researchallofus.org/ehr/labs-and-measurements/36660924) | LOINC | LP419289-6 | 3,740 | 0 |
| SARS-CoV-2 (COVID-19) S protein RBD neutralizing antibody [Presence] in Serum or Plasma by sVNT | [36031734](https://databrowser.researchallofus.org/ehr/labs-and-measurements/36031734) | LOINC | 96603-6 | 0 | 476 |
| SARS-CoV-2 (COVID-19) sequencing and identification panel | [1620099](https://databrowser.researchallofus.org/ehr/labs-and-measurements/1620099) | LOINC | LP422740-3 | 40 | 0 |
| SARS-CoV-2 (COVID-19) sequencing and identification panel - Specimen by Molecular genetics method | [36033651](https://databrowser.researchallofus.org/ehr/labs-and-measurements/36033651) | LOINC | 96894-1 | 0 | 40 |
| SARS-CoV-2 (COVID-19) sequencing and identification panel \| XXX \| Microbiology Panels | [1618441](https://databrowser.researchallofus.org/ehr/labs-and-measurements/1618441) | LOINC | LP427524-6 | 40 | 0 |
| SARS-CoV-2 (COVID-19) spike protein receptor binding domain (RBD) | [36033856](https://databrowser.researchallofus.org/ehr/labs-and-measurements/36033856) | LOINC | LP421235-5 | 476 | 0 |
| SARS-CoV-2 (COVID-19) spike protein receptor binding domain (RBD) neutralizing antibody | [36033858](https://databrowser.researchallofus.org/ehr/labs-and-measurements/36033858) | LOINC | LP421234-8 | 476 | 0 |
| SARS-CoV-2 (COVID-19) spike protein receptor binding domain (RBD) neutralizing antibody \| Serum or Plasma \| Microbiology | [36033625](https://databrowser.researchallofus.org/ehr/labs-and-measurements/36033625) | LOINC | LP421840-2 | 476 | 0 |
| SARS-CoV+SARS-CoV-2 (COVID-19) | [36661687](https://databrowser.researchallofus.org/ehr/labs-and-measurements/36661687) | LOINC | LP418774-8 | 1,075 | 0 |
| SARS-CoV+SARS-CoV-2 (COVID-19) Ag | [36661520](https://databrowser.researchallofus.org/ehr/labs-and-measurements/36661520) | LOINC | LP418762-3 | 1,075 | 0 |
| SARS-CoV+SARS-CoV-2 (COVID-19) Ag [Presence] in Respiratory specimen by Rapid immunoassay | [757685](https://databrowser.researchallofus.org/ehr/labs-and-measurements/757685) | LOINC | 95209-3 | 0 | 1,075 |
| SARS-CoV+SARS-CoV-2 (COVID-19) Ag \| Respiratory specimen \| Microbiology | [1618075](https://databrowser.researchallofus.org/ehr/labs-and-measurements/1618075) | LOINC | LP427654-1 | 1,075 | 0 |
| **COVID-19 Participant Experience (COPE) survey response** |  |  |  |  |  |
| COPE Survey (Any version): In the past month, have you been sick for more than one day with a new illness related to COVID-19 or flu-like symptoms?: Yes | 1332898 |  |  | 9,138 |  |
| **Diagnostic codes** |  |  |  |  |  |
| COVID-19 | [37311061](https://databrowser.researchallofus.org/ehr/conditions/37311061) | SNOMED | 840539006 | 17,384 | 17,384 |
| Lower respiratory infection caused by SARS-CoV-2 | [3663281](https://databrowser.researchallofus.org/ehr/conditions/3663281) | SNOMED | 8.8053E+17 | 1,663 | 0 |

Table A.1. Caption: All laboratory observations, COVID-19 Participant Experience (COPE) survey item responses, and diagnostic codes in SNOMED vocabulary indicating either SARS-CoV-2 infection or COVID-19 illness. Generated using queries for lab values, survey item responses, and diagnostic code incidences via the All of Us Researcher Workbench dataset builder.
