# Supplementary material for: Long COVID risk by pre-infection symptoms and functional status: A retrospective cohort study of data from the All of Us Research Program
Source: PLoS One. 2026 Jun 16;21(6):e0330793. doi: 10.1371/journal.pone.0330793 (PMC13271467; doi:10.1371/journal.pone.0330793)
Supplement: S3 Table — Standard concept names, concept codes, source vocabularies, and included sub-concepts of long COVID symptoms queried via the All of Us Researcher Workbench dataset builder. Hierarchical relationships are as organized in the Athena relational database of the Observational Health Data Sciences and Informatics (OHDSI) and queried from the Observational Medical Outcomes Partnership – Common Data Model (OMOP – CDM) table structures. (DOCX) [file pone.0330793.s003.docx]

**Table A.2. Long COVID symptoms/conditions used in Cohort discovery and classification.**

| **Standard Concepts** | **OMOP Concept ID** | **Source** | **Vocab** | **Code** | **Strings collapsed** |
| --- | --- | --- | --- | --- | --- |
| Abdominal pain | [200219](https://databrowser.researchallofus.org/ehr/conditions/200219) | Standard | SNOMED | 21522001 | "abdominal" |
| Anxiety | [441542](https://databrowser.researchallofus.org/ehr/conditions/441542) | Standard | SNOMED | 48694002 | "anxiety", "anxi*" |
| Chest pain | [77670](https://databrowser.researchallofus.org/ehr/conditions/77670) | Standard | SNOMED | 29857009 | "chest", "cardiac" |
| Chronic fatigue syndrome | [432738](https://databrowser.researchallofus.org/ehr/conditions/432738) | Standard | SNOMED | 52702003 | "chronic fatigue", "myalgic encephalomyelitis" |
| Cognitive disorder  (collapsed into “impaired cognition”) | [40480615](https://databrowser.researchallofus.org/ehr/conditions/40480615) | Standard | SNOMED | 443265004 | "cognitive", "neurocognitive" |
| Cognitive function finding (collapsed into “impaired cognition”) | [4162723](https://databrowser.researchallofus.org/ehr/conditions/4162723) | Standard | SNOMED | 373930000 | [not collapsed, heterogeneous] |
| Cough | [254761](https://databrowser.researchallofus.org/ehr/conditions/254761) | Standard | SNOMED | 49727002 | "cough", "clearing throat" |
| Depression screening positive | [762504](https://databrowser.researchallofus.org/ehr/conditions/762504) | Standard | SNOMED | 4.28181E+14 | "depression", "screening" |
| Depressive disorder | [440383](https://databrowser.researchallofus.org/ehr/conditions/440383) | Standard | SNOMED | 35489007 | "depressive", "depression" |
| Depressive episode | [3656234](https://databrowser.researchallofus.org/ehr/conditions/3656234) | Standard | SNOMED | 871840004 |  |
| Diarrhea | [196523](https://databrowser.researchallofus.org/ehr/conditions/196523) | Standard | SNOMED | 62315008 | "diarrhea", "diarrheal" |
| Disorder of menstruation | [443431](https://databrowser.researchallofus.org/ehr/conditions/443431) | Standard | SNOMED | 386804004 | "*men* |
| Disturbance in sleep behavior (collapsed into “Sleep”) | [4204989](https://databrowser.researchallofus.org/ehr/conditions/4204989) | Standard | SNOMED | 53888004 | "sleep" |
| Dizziness | [4223938](https://databrowser.researchallofus.org/ehr/conditions/4223938) | Standard | SNOMED | 404640003 | "dizziness", "dizzy", "vertigo" |
| Dyspnea | [312437](https://databrowser.researchallofus.org/ehr/conditions/312437) | Standard | SNOMED | 267036007 | "dyspnea", "*pnea", "gasping" |
| Eruption | [140214](https://databrowser.researchallofus.org/ehr/conditions/140214) | Standard | SNOMED | 271807003 | "eruption", "acne*", "rash", "eruption", "erythroderma", "exanthematous", "Fox-Fordyce", "rocacea", "Keratin*", "pityriasis", "psoriasis", "psoriatic", "dermatitis" |
| Fatigue | [4223659](https://databrowser.researchallofus.org/ehr/conditions/4223659) | Standard | SNOMED | 84229001 | "asthenia" |
| Fever | [437663](https://databrowser.researchallofus.org/ehr/conditions/437663) | Standard | SNOMED | 386661006 | "fever", "*pyrexia" |
| Finding of pattern of menstrual cycle (collapsed into “menstrual disorder”) | 4095940 | Standard | SNOMED | 248968007 | "menstru*", "amenorrhea" |
| Finding of sexual function | [4041277](https://databrowser.researchallofus.org/ehr/conditions/4041277) | Standard | SNOMED | 118202007 |  |
| Headache | [378253](https://databrowser.researchallofus.org/ehr/conditions/378253) | Standard | SNOMED | 25064002 | "headache", "head" |
| Impaired Cognition | [443432](https://databrowser.researchallofus.org/ehr/conditions/443432) | Standard | SNOMED | 386806002 | "cognitive", "impairment", "behavioral" |
| Irregular periods (collapsed into “menstrual disorder”) | [196168](https://databrowser.researchallofus.org/ehr/conditions/196168) | Standard | SNOMED | 80182007 |  |
| Joint pain | [77074](https://databrowser.researchallofus.org/ehr/conditions/77074) | Standard | SNOMED | 57676002 | "arthralgia", "joint" |
| Lightheadedness | [4297376](https://databrowser.researchallofus.org/ehr/conditions/4297376) | Standard | SNOMED | 386705008 | "malaise and fatigue", "fatigue".  Collapsed into “Fatigue” |
| Loss of sense of smell | [4185711](https://databrowser.researchallofus.org/ehr/conditions/4185711) | Standard | SNOMED | 44169009 |  |
| Loss of taste | [4289517](https://databrowser.researchallofus.org/ehr/conditions/4289517) | Standard | SNOMED | 36955009 |  |
| Malaise (under “Lightheadedness” heirarchy) | 4272240 | Standard | SNOMED | 367391008 |  |
| Muscle fatigue | [4214612](https://databrowser.researchallofus.org/ehr/conditions/4214612) | Standard | SNOMED | 80449002 | "muscle" |
| Muscle pain | [442752](https://databrowser.researchallofus.org/ehr/conditions/442752) | Standard | SNOMED | 68962001 | "myalgia*", "pain", "muscle", "pleurodynia", "fibrositis", "fibromyalgia", "polymyalgia", "claudication", "migraine" |
| Musculoskeletal chest pain | [4092930](https://databrowser.researchallofus.org/ehr/conditions/4092930) | Standard | SNOMED | 281245003 | "pleurodynia", "myalgia", "pain", "Scapulalgia", "Xiphodynia", "syndrome" |
| Palpitations | 315078 | Standard | SNOMED | 80313002 | "heart", "palpitations" |
| Paraesthesia | [4236484](https://databrowser.researchallofus.org/ehr/conditions/4236484) | Standard | SNOMED | 91019004 | ""*esthesia", "sensation", "pins", |
| Post-acute COVID-19 | [705076](https://databrowser.researchallofus.org/ehr/conditions/705076) | Standard | OMOP Extension | OMOP5160861 | U09.9 |
| Postural orthostatic tachycardia syndrome (POTS) | [4159659](https://databrowser.researchallofus.org/ehr/conditions/orthostatic%20tachycardia) |  | SNOMED | 371073003 | (No hierarchy) |
| Postviral fatigue syndrome | [4202045](https://databrowser.researchallofus.org/ehr/conditions/4202045) | Standard | SNOMED | 51771007 |  |
| Sleep disorder (collapsed into “Sleep”) | [435524](https://databrowser.researchallofus.org/ehr/conditions/435524) | Standard | SNOMED | 39898005 | "sleep*", "dream", "*somnia", "somnolence" |
| Tachycardia | [444070](https://databrowser.researchallofus.org/ehr/conditions/444070) | Standard | SNOMED | 3424008 | "tachycardia*" |

Table A.2. Caption: Standard concept names, concept codes, source vocabularies, and included sub-concepts of long COVID symptoms queried via the *All of Us* Researcher Workbench dataset builder. Hierarchical relationships are as organized in the Athena relational database of the Observational Health Data Sciences and Informatics (OHDSI) and queried from the Observational Medical Outcomes Partnership – Common Data Model (OMOP – CDM) table structures.
