# Supplementary material for: Long COVID risk by pre-infection symptoms and functional status: A retrospective cohort study of data from the All of Us Research Program
Source: PLoS One. 2026 Jun 16;21(6):e0330793. doi: 10.1371/journal.pone.0330793 (PMC13271467; doi:10.1371/journal.pone.0330793)
Supplement: S4 Table — Standard concept names, codes, vaccine type, and counts of vaccination data queried in All of Researcher Workbench dataset builder, Drug Encounters OMOP Table. Raw counts are from the unfiltered (N = 104,993) sample of every vaccination encounter. (DOCX) [file pone.0330793.s004.docx]

**Table A.3. Vaccination type and counts**

| **Standard Concepts** | **Standard Concept Code** | **type** | ***n*** |
| --- | --- | --- | --- |
| SARS-CoV-2 (COVID-19) vaccine, mRNA-BNT162b2 0.1 MG/ML Injectable Suspension | 2468235 | mRNA | 17485 |
| SARS-CoV-2 (COVID-19) vaccine, mRNA-1273 0.2 MG/ML Injectable Suspension | 2470234 | mRNA | 8802 |
| SARS-CoV-2 (COVID-19) vaccine, mRNA spike protein | 2468231 | mRNA | 1084 |
| SARS-COV-2 (COVID-19) vaccine, mRNA, spike protein, LNP, preservative free, 30 mcg/0.3mL dose, tris-sucrose formulation | 217 | mRNA | 985 |
| SARS-COV-2 (COVID-19) vaccine, vector - Ad26 100000000000 UNT/ML Injectable Suspension | 2479835 | protein or vector | 984 |
| SARS-CoV-2 (COVID-19) vaccine, mRNA-1273 0.2 MG/ML | 2470233 | mRNA | 230 |
| SARS-COV-2 (COVID-19) vaccine, vector non-replicating, recombinant spike protein-ChAdOx1, preservative free, 0.5 mL | 210 | protein or vector | 22 |
| SARS-COV-2 (COVID-19) vaccine, vector non-replicating | 2479831 | protein or vector | 18 |
| SARS-COV-2 (COVID-19) vaccine, UNSPECIFIED | 213 | protein or vector | 17 |
|  | OMOP5048605 | protein or vector | 1 |
| SARS-COV-2 (COVID-19) vaccine, mRNA, spike protein, LNP, preservative free, 10 mcg/0.2mL dose, tris-sucrose formulation | 218 | mRNA | 1 |
| SARS-COV-2 COVID-19 Inactivated Virus Non-US Vaccine Product (BIBP, Sinopharm) | 510 | protein or vector | 1 |
| SARS-CoV-2 (COVID-19) vaccine, mRNA spike protein Injectable Suspension | 2468234 | mRNA | 1 |

Table A.3. Caption: Standard concept names, codes, vaccine type, and counts of vaccination data queried in All of Researcher Workbench dataset builder, Drug Encounters OMOP Table. Raw counts are from the unfiltered (N=104,993) sample of every vaccination encounter.
