# Supplementary material for: Long COVID risk by pre-infection symptoms and functional status: A retrospective cohort study of data from the All of Us Research Program
Source: PLoS One. 2026 Jun 16;21(6):e0330793. doi: 10.1371/journal.pone.0330793 (PMC13271467; doi:10.1371/journal.pone.0330793)
Supplement: S5 Fig — Jitter point horizontal arrays showing the overlapping ranges in propensity for being in the long COVID group between the unmatched versus matched pre-infection enrollees (‘control’) and post-infection enrollees (‘treated’; excluded for the present study’s analysis). After matching pre- and post-enrollment participants, about a 45% overlap is seen between these two groups, indicating that participants may differ in one or more key demographic and disease aspects relating to when they enrolled in the study. (DOCX) [file pone.0330793.s005.docx]

**Fig C.1. Distribution of propensity scores for pre- versus post-infection enrollment.**


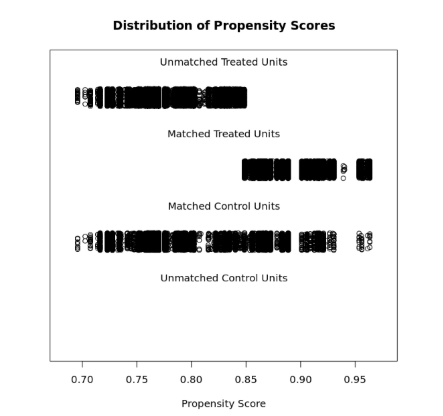


Table C.1. Caption: Jitter point horizontal arrays showing the overlapping ranges in propensity for being in the long COVID group between the unmatched versus matched pre-infection enrollees (‘control’) and post-infection enrollees (‘treated’; excluded for the present study’s analysis). After matching pre- and post-enrollment participants, about a 45% overlap is seen between these two groups, indicating that participants may differ in one or more key demographic and disease aspects relating to when they enrolled in the study.
