# Supplementary material for: Long COVID risk by pre-infection symptoms and functional status: A retrospective cohort study of data from the All of Us Research Program
Source: PLoS One. 2026 Jun 16;21(6):e0330793. doi: 10.1371/journal.pone.0330793 (PMC13271467; doi:10.1371/journal.pone.0330793)
Supplement: S6 Fig — Differences between standardized means of variant, race, sex, and education between participants who joined All of Us before versus after their first infection. Large differences are observed in race and education, with an overall distance of about 0.2 between these groups’ means. These demographic differences most likely reflect period differences in study enrollment efforts targeting under-represented groups in medical research, and may indicate that some of these groups are underrepresented in the present sample. (DOCX) [file pone.0330793.s006.docx]

**Fig C.2. Standardized Mean Difference of demographic and disease characteristics by pre- versus post-infection enrollment.**


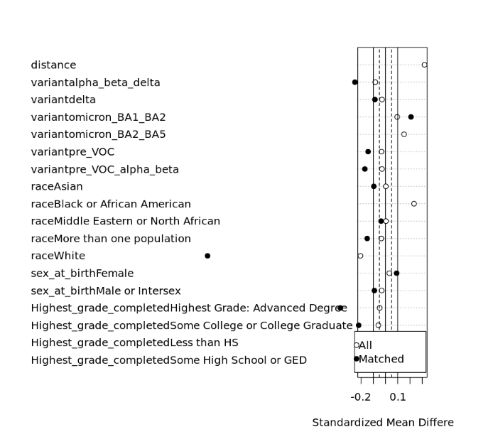


Fig. C.2. Caption: Differences between standardized means of variant, race, sex, and education between participants who joined *All of Us* before versus after their first infection. Large differences are observed in race and education, with an overall distance of about 0.2 between these groups’ means. These demographic differences most likely reflect period differences in study enrollment efforts targeting under-represented groups in medical research, and may indicate that some of these groups are underrepresented in the present sample.
