# Supplementary material for: Long COVID risk by pre-infection symptoms and functional status: A retrospective cohort study of data from the All of Us Research Program
Source: PLoS One. 2026 Jun 16;21(6):e0330793. doi: 10.1371/journal.pone.0330793 (PMC13271467; doi:10.1371/journal.pone.0330793)
Supplement: S7 Fig — Overlaps in propensity score between the participants classified as cases at 28 days (‘control’) and those at 90 days (‘treated’) is visualized in Fig C.3. There was no overlap between cases and controls by the 90-day alternative classification scheme (distance = 1.0). (DOCX) [file pone.0330793.s007.docx]

**Fig. C.3. Distribution of propensity scores for 28-day versus 90-day symptom onset date.**


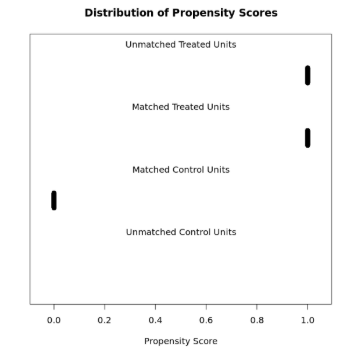


Fig. C.3. Caption: Overlaps in propensity score between the participants classified as cases at 28 days (‘control’) and those at 90 days (‘treated’) is visualized in Figure S.C.3. There was no overlap between cases and controls by the 90-day alternative classification scheme (distance = 1.0).
