# Supplementary material for: Long COVID risk by pre-infection symptoms and functional status: A retrospective cohort study of data from the All of Us Research Program
Source: PLoS One. 2026 Jun 16;21(6):e0330793. doi: 10.1371/journal.pone.0330793 (PMC13271467; doi:10.1371/journal.pone.0330793)
Supplement: S8 Fig — Plot showing the difference in standardized mean propensity for classification as a long COVID case according to either a 28 day or a 90 day lag from first infection indicator. The matched samples show no significant difference in classification likelihood, indicating that, in this sample and study design, classifying participants as “cases” who had their first long COVID symptom 28 days or more after infection did not result in a significantly different case-versus-control distribution than classifying based on first symptom at 90 days or more. (DOCX) [file pone.0330793.s008.docx]

**Fig. C.4. Standardized Mean Difference of demographic and disease characteristics by 28-day versus 90-day symptom onset date**


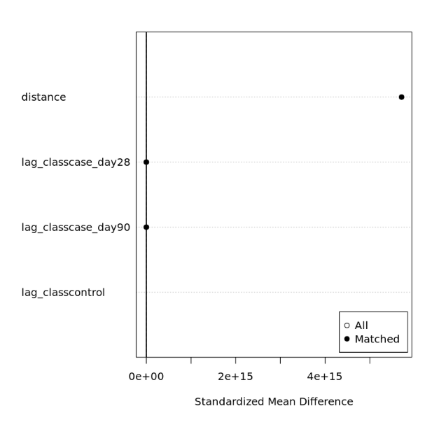


Fig C.4. Caption: Plot showing the difference in standardized mean propensity for classification as a long COVID case according to either a 28 day or a 90 day lag from first infection indicator. The matched samples show no significant difference in classification likelihood, indicating that, in this sample and study design, classifying participants as “cases” who had their first long COVID symptom 28 days or more after infection did not result in a significantly different case-versus-control distribution than classifying based on first symptom at 90 days or more.
