# Supplementary material for: Long COVID risk by pre-infection symptoms and functional status: A retrospective cohort study of data from the All of Us Research Program
Source: PLoS One. 2026 Jun 16;21(6):e0330793. doi: 10.1371/journal.pone.0330793 (PMC13271467; doi:10.1371/journal.pone.0330793)
Supplement: S9 Table — Demographic, disease, pre-infection symptom, and pre-infection function variables (names, sources, and formats) used as covariates in regression models. Pre-infection variables are based on entry in the medical record between five years and four weeks prior to first infection date. Each variable’s intercept value or level is noted. (DOCX) [file pone.0330793.s009.docx]

**Table D.1. Variables for model.**

| **Type** | **Covariate** | **Sources** | **Model index level/value**** |
| --- | --- | --- | --- |
| Demographics | Age  Sex at birth  Race  Ethnicity | Self-reported “Basics” survey responses at enrollment  EMR | Age = **62**  Female or intersex  White  Not Hispanic/Latino |
| Acquired demographics/SDH | Education (highest level completed) | Self-reported “Basics” survey responses | Some College |
| SARS-CoV-2 Variant | The calendar period between the starts of major variants of concern in which the first infection occurred. | Self-report of symptoms on the COPE surveys  EMR condition and measurement codes (SNOMED, OMOP, and ICD-10 vocabularies). | Pre-VOC period |
| Vaccination with full series** | Binary indicator of “full series” or “not vaccinated” according to the CDC’s definition at the time of data cut-off (at least a two-dose primary series of mRNA or at least one dose of all other types).  To determine optimal vaccination data structure, the R^2^, AIC, and BIC model fit statistics were compared for four versions of the final model with vaccination fitted in one of four ways:   1. Month/year datetime 2. Lag time in days between first primary series and first infection 3. Vaccination before vs. after first infection. 4. Binary (“full series” versus “not vaccinated”).   Due to the small number of people with vaccination at the time of this dataset’s cut-off, models a-c were overparameterized against the primary covariate of functional status, and were thus severely overfitted.  The best fit to these data was found for the binary predictor (model d). | Drug codes | Not vaccinated |
| Pre-infection health and symptoms | Pre-infection total number of long COVID symptom categories with at least one incidence*  Self-Reported mental health and cognition  Self-Reported ability to perform physically-demanding daily activities  Self-Reported social role performance and satisfaction (composite score summing two ordinal items, values 2(~excellent)-10(~very poor) | Self-reported “Overall Health” survey responses at enrollment  EMR condition codes (SNOMED, OMOP, and ICD-10 vocabularies). | Total number of symptoms with any pre-infection occurrence = **4** (median)  Individual symptom categories = **0** (no occurrences) |
| Pre-infection daily Functioning | Pre-infection total number of occupational therapy CPT codes*  Incidences of at least one pre-infection functional performance finding diagnostic codes*, collapsed into three levels. | EMR condition and procedure codes (SNOMED, OMOP, and ICD-10 vocabularies). | CPT total = **0**  “Finding of Functional Performance” impairment level = **“None”** |
| Note. * = Entered in EHR between five years and four weeks before first infection date. ** = Mean, median, or most frequently observed categorical level.  ** = Full primary series as defined by [CDC National Healthcare Safety Network Up To Date Guidance for surveillance](https://www.cdc.gov/nhsn/pdfs/hps/covidvax/UpToDateGuidance-508.pdf) at the time of *All of* Us CDR 7.0 July 1, 2022 cut-off (June - September 2022). | | | |

Table D. 1. Caption: Demographic, disease, pre-infection symptom, and pre-infection function variables (names, sources, and formats) used as covariates in regression models. Pre-infection variables are based on entry in the medical record between five years and four weeks prior to first infection date. Each variable’s intercept value or level is noted.
