# Supplementary material for: Long COVID risk by pre-infection symptoms and functional status: A retrospective cohort study of data from the All of Us Research Program
Source: PLoS One. 2026 Jun 16;21(6):e0330793. doi: 10.1371/journal.pone.0330793 (PMC13271467; doi:10.1371/journal.pone.0330793)
Supplement: S10 Table — The final model had 78 degrees of freedom from the fitted parameters. Model discrimination was good, with a maximum likelihood ratio X2 of 26194.58 and an area under the receiver operating curve (C) of 85%. The R2 – total variance explained by the fitted model – was 45%, indicating about half of the variance was unexplained by this model. Given the large sample size and the highly significant X2 of the maximum likelihood ratio (p < 0.0001), this model was judged to have adequate fit for determining association. (DOCX) [file pone.0330793.s010.docx]

**Table D.2. Model summary**

|  | Model Likelihood Ratio Test | Discrimination  Indexes | Rank Discrim.  Indexes |
| --- | --- | --- | --- |
| Obs 65464 | LR chi2 26194.58 | *R^2^*  0.449 | C 0.850 |
| FALSE 24809 | d.f. 78 | *R^2^* (78,65464)0.329 | Dxy 0.699 |
| TRUE 40655 | Pr(> chi2) <0.0001 | *R^2^* (78,46221.3)0.432 | gamma 0.699 |
| max \|deriv\| 3e-08 | | Brier 0.150 | tau-a 0.329 |

Table D.2. Caption: The final model had 78 degrees of freedom from the fitted parameters. Model discrimination was good, with a maximum likelihood ratio *X^2^* of 26194.58 and an area under the receiver operating curve (C) of 85%. The *R^2^* – total variance explained by the fitted model – was 45%, indicating about half of the variance was unexplained by this model. Given the large sample size and the highly significant *X^2^* of the maximum likelihood ratio (*p* <0.0001), this model was judged to have adequate fit for determining association.
