# Supplementary material for: Long COVID risk by pre-infection symptoms and functional status: A retrospective cohort study of data from the All of Us Research Program
Source: PLoS One. 2026 Jun 16;21(6):e0330793. doi: 10.1371/journal.pone.0330793 (PMC13271467; doi:10.1371/journal.pone.0330793)
Supplement: S11 Fig — Plot showing the contribution of each variable (X2) to the model’s omnibus effect size. Covariates are plotted on the Y axis; the least-significant contributor is at the top, and the rest are plotted down the axis in order of effect to the most-significant contributor at the bottom. The effect size is plotted on the X axis as X2 minus the degrees of freedom. Each variable’s effect is plotted as a dot intersecting the covariate with its effect size value. The top (smallest) contributor is the interaction between age and prior functional performance (X2 = 0.0, p = 0.994); the bottom (largest) contributor is infection variant, (X2 = 5832.6, p < 0.0000). (DOCX) [file pone.0330793.s011.docx]

**Fig D.1. Contribution of each variable to model effects**


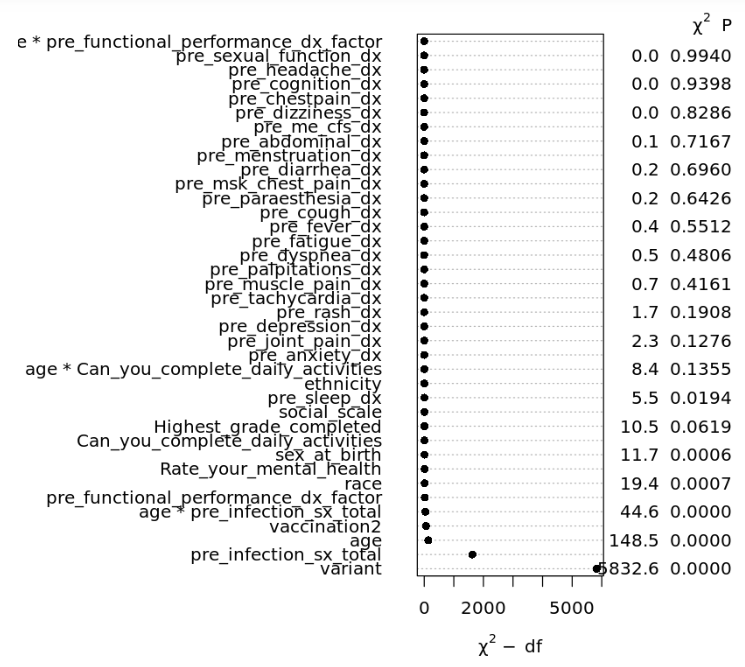


Figure D.1. Caption: Plot showing the contribution of each variable (X2) to the model’s omnibus effect size. Covariates are plotted on the Y axis; the least-significant contributor is at the top, and the rest are plotted down the axis in order of effect to the most-significant contributor at the bottom. The effect size is plotted on the X axis as X2 minus the degrees of freedom. Each variable’s effect is plotted as a dot intersecting the covariate with its effect size value. The top (smallest) contributor is the interaction between age and prior functional performance (X2=0.0, p=0.994); the bottom (largest) contributor is infection variant, (X2=5832.6, p<0.0000).
