# Supplementary material for: Long COVID risk by pre-infection symptoms and functional status: A retrospective cohort study of data from the All of Us Research Program
Source: PLoS One. 2026 Jun 16;21(6):e0330793. doi: 10.1371/journal.pone.0330793 (PMC13271467; doi:10.1371/journal.pone.0330793)
Supplement: S12 Table — Variance inflation factor (VIF) for the parameters of each covariate, an estimate of multicollinearity in the model. Variance inflation and collinearity can result from several covariates’ effects on the outcome increasing or decreasing at the same rate and/or magnitude (such that the effect of one could be predicted by or accounted for by the other). VIF was below 5.0 for most covariates, indicating low risk for multicollinearity. Seventeen parameter values (e.g., levels in a categorical variable or interaction) were above 5. None exceeded 10. (DOCX) [file pone.0330793.s012.docx]

**Table D.4. Variance inflation factor for each covariate**

| **Covariate** | **Variance inflation factor** |
| --- | --- |
|  |  |
| variant=Pre-VOC, Alpha, Beta | 1.238834e+00 |
| variant=Alpha, Beta, Delta | 1.132717e+00 |
| variant=Delta | 1.176021e+00 |
| variant=Omicron BA1-BA2 | 1.200145e+00 |
| variant=Omicron BA2-BA5 | 1.113494e+00 |
| vaccination=Full Series | 1.021039e+00 |
| Age | 6.049463e+00 |
| sex_at_birth=Male | 1.128947e+00 |
| race=Black or African American | 5.895579e+00 |
| race=Middle Eastern or North African | 1.212751e+00 |
| race=More than one population | 1.583254e+00 |
| race=White | 6.164593e+00 |
| ethnicity=Not Hispanic or Latino | 1.019674e+00 |
| Highest_grade_completed=College or college grad | 1.494630e+00 |
| Highest_grade_completed=High School/GED | 1.570400e+00 |
| Highest_grade_completed=Some High School | 1.229526e+00 |
| Highest_grade_completed=Less than high school | 1.044473e+00 |
| Highest_grade_completed=No Answer | 1.060842e+00 |
| pre_infection_sx_total | 4.144329e+03 |
| pre_infection_sx_total' | 6.960308e+04 |
| pre_infection_sx_total'' | 1.413850e+05 |
| pre_infection_sx_total''' | 1.974925e+04 |
| social_scale=3 | 1.831219e+00 |
| social_scale=4 | 2.475786e+00 |
| social_scale=5 | 2.278675e+00 |
| social_scale=6 | 2.530618e+00 |
| social_scale=7 | 1.994741e+00 |
| social_scale=8 | 1.915202e+00 |
| social_scale=9 | 1.449554e+00 |
| social_scale=10 | 1.351644e+00 |
| Rate_your_mental_health=Very Good | 2.083809e+00 |
| Rate_your_mental_health=Good | 2.512260e+00 |
| Rate_your_mental_health=Fair | 2.290396e+00 |
| Rate_your_mental_health=Poor | 1.508990e+00 |
| Rate_your_mental_health=No Answer | 1.051157e+00 |
| Can_you_complete_daily_activities=Mostly | 1.490209e+01 |
| Can_you_complete_daily_activities=Moderately | 1.720550e+01 |
| Can_you_complete_daily_activities=A little | 2.072651e+01 |
| Can_you_complete_daily_activities=Not at all | 1.985066e+01 |
| Can_you_complete_daily_activities=No Answer | 1.777763e+01 |
| pre_functional_performance_dx_factor=Some | 3.912027e+01 |
| pre_functional_performance_dx_factor=Dependent | 3.544598e+01 |
| cpt_total_precovid | 1.052716e+00 |
| pre_abdominal_dx | 6.944247e+01 |
| pre_anxiety_dx | 6.221869e+01 |
| pre_chestpain_dx | 6.856771e+01 |
| pre_cough_dx | 5.960201e+01 |
| pre_depression_dx | 5.786623e+01 |
| pre_diarrhea_dx | 3.670505e+01 |
| pre_dizziness_dx | 4.089403e+01 |
| pre_dyspnea_dx | 5.324548e+01 |
| pre_fatigue_dx | 5.307157e+01 |
| pre_fever_dx | 2.560118e+01 |
| pre_headache_dx | 4.415449e+01 |
| pre_cognition_dx | 2.132864e+01 |
| pre_joint_pain_dx | 8.048326e+01 |
| pre_me_cfs_dx | 9.517710e+00 |
| pre_menstruation_dx | 2.559214e+01 |
| pre_muscle_pain_dx | 2.436233e+01 |
| pre_msk_chest_pain_dx | 1.347388e+01 |
| pre_palpitations_dx | 2.703584e+01 |
| pre_paraesthesia_dx | 2.203656e+01 |
| pre_sexual_function_dx | 5.349296e+00 |
| pre_rash_dx | 4.722936e+01 |
| pre_sleep_dx | 6.044857e+01 |
| pre_tachycardia_dx | 2.275191e+01 |
| age * Can_you_complete_daily_activities=Mostly | 1.525799e+01 |
| age * Can_you_complete_daily_activities=Moderately | 1.730822e+01 |
| age * Can_you_complete_daily_activities=A little | 2.043648e+01 |
| age * Can_you_complete_daily_activities=Not at all | 1.979777e+01 |
| age * Can_you_complete_daily_activities=No Answer | 1.778441e+01 |
| age * pre_functional_performance_dx_factor=Some | 2.192163e+01 |
| age * pre_functional_performance_dx_factor=Dependent | 2.715921e+01 |
| age * pre_infection_sx_total | 8.839911e+02 |
| age * pre_infection_sx_total' | 7.293169e+04 |
| age * pre_infection_sx_total'' | 1.456008e+05 |
| age * pre_infection_sx_total''' | 1.999367e+04 |

Table D.4. Caption: Variance inflation factor (VIF) for the parameters of each covariate, an estimate of multicollinearity in the model. Variance inflation and collinearity can result from several covariates’ effects on the outcome increasing or decreasing at the same rate and/or magnitude (such that the effect of one could be predicted by or accounted for by the other). VIF was below 5.0 for most covariates, indicating low risk for multicollinearity. Seventeen parameter values (e.g. levels in a categorical variable or interaction) were above 5. None exceeded 10.
