# Supplementary material for: Long COVID risk by pre-infection symptoms and functional status: A retrospective cohort study of data from the All of Us Research Program
Source: PLoS One. 2026 Jun 16;21(6):e0330793. doi: 10.1371/journal.pone.0330793 (PMC13271467; doi:10.1371/journal.pone.0330793)
Supplement: S13 Fig — Histogram of the number of participants with (darker blue) versus without (lighter blue) long COVID by first infection date through July 2022. The Y axis plots the number of participants ascending from zero (bottom) to over 1,500 (top); the X axis is a timeline from (left to right) January 1 2020 through July 31 2022. Both groups show a similar profile; they begin with a steep spike in infections about March 2020, a large and prolonged peak between April and September 2020, and briefer peaks between about October 2020 – January 2021 and in January 2022. (DOCX) [file pone.0330793.s013.docx]

**Fig. E.1. Time of first infection, by long COVID group.**


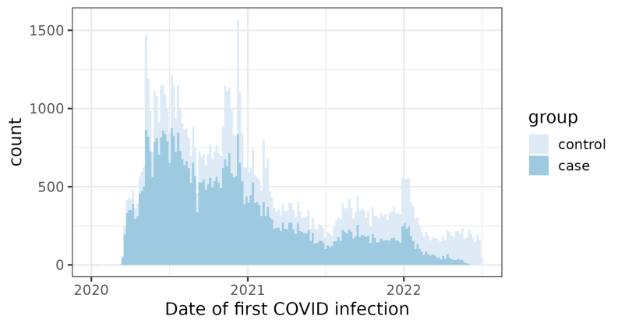


Fig. E.1. Caption: Histogram of the number of participants with (darker blue) versus without (lighter blue) long COVID by first infection date through July 2022. The *Y* axis plots the number of participants ascending from zero (bottom) to over 1,500 (top); the *X* axis is a timeline from (left to right) January 1 2020 through July 31 2022. Both groups show a similar profile; they begin with a steep spike in infections about March 2020, a large and prolonged peak between April and September 2020, and briefer peaks between about October 2020 – January 2021 and in January 2022.
