# Supplementary material for: Long COVID risk by pre-infection symptoms and functional status: A retrospective cohort study of data from the All of Us Research Program
Source: PLoS One. 2026 Jun 16;21(6):e0330793. doi: 10.1371/journal.pone.0330793 (PMC13271467; doi:10.1371/journal.pone.0330793)
Supplement: S14 Fig — Two side-by-side bar graphs (controls on the left, cases on the right) of the timing of first infection and first full-series vaccination among the n = 3,210 participants who were fully vaccinated. This graph suggests that, among participants with full vaccination, those with at least one long COVID symptom at any point had a higher disease incidence through early 2022 (spiking sharply in early 2020 and tapering gradually through 2021), and a higher uptake of both initial vaccination (December 2020 through about June 2021) and boosters (spiking in October 2021 and April 2022). (DOCX) [file pone.0330793.s014.docx]

**Fig. E.2. Among vaccinated participants, distributions of first infection and first vaccination date.**


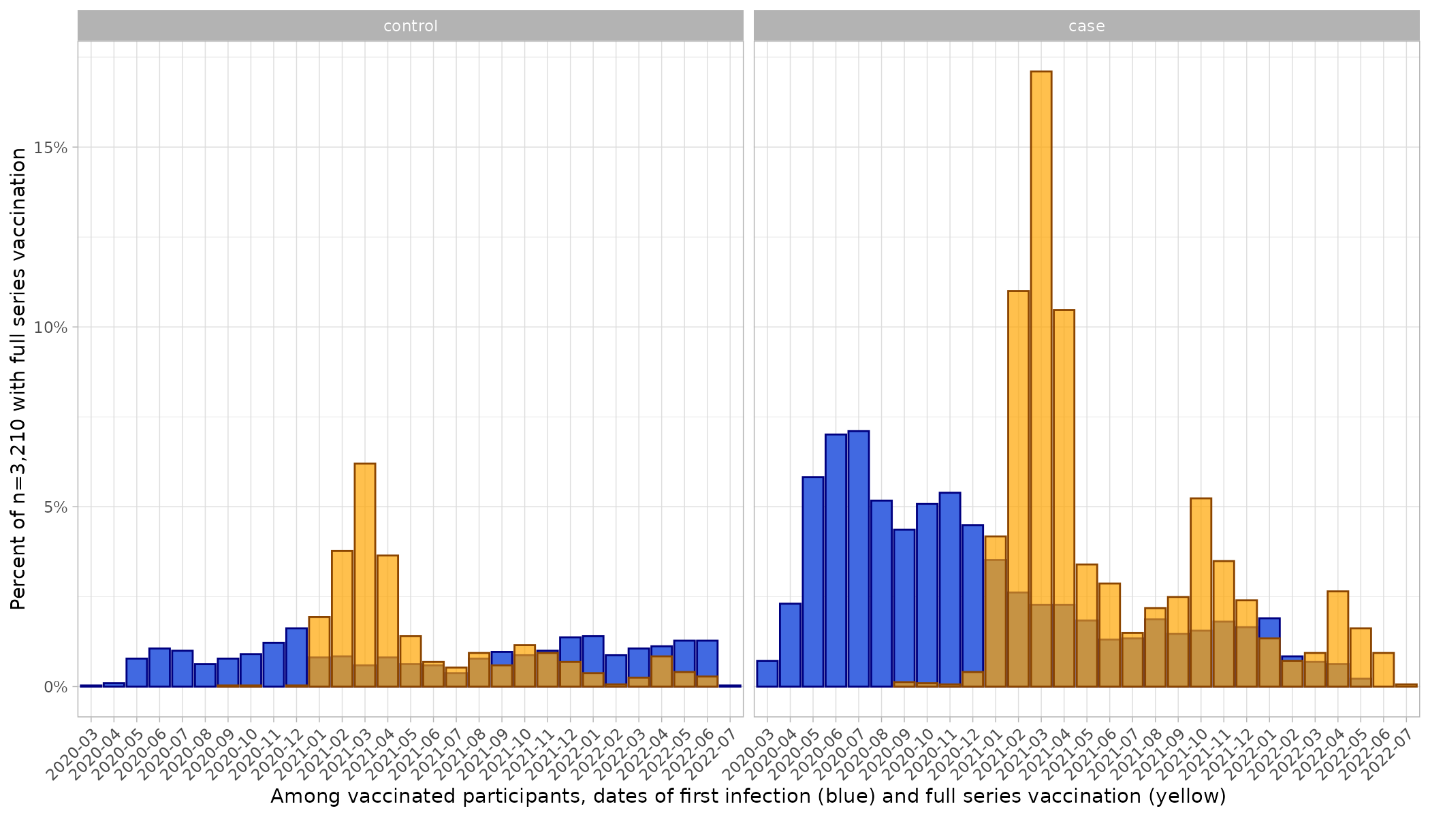


Fig. E.2. Caption: Two side-by-side bar graphs (controls on the left, cases on the right) of the timing of first infection and first full-series vaccination among the n=3,210 participants who were fully vaccinated. This graph suggests that, among participants with full vaccination, those with at least one long COVID symptom at any point had a higher disease incidence through early 2022 (spiking sharply in early 2020 and tapering gradually through 2021), and a higher uptake of both initial vaccination (December 2020 through about June 2021) and boosters (spiking in October 2021 and April 2022).
