# Supplementary material for: Long COVID risk by pre-infection symptoms and functional status: A retrospective cohort study of data from the All of Us Research Program
Source: PLoS One. 2026 Jun 16;21(6):e0330793. doi: 10.1371/journal.pone.0330793 (PMC13271467; doi:10.1371/journal.pone.0330793)
Supplement: S15 Tables — Interactions between Age and pre-infection EHR functional status observations, self-reported physical activity, and total incident symptoms. E.1. Adjusted odds by age and pre-infection functional performance level (EHR code under “Finding of Functional Performance and Activity”) of developing long COVID. E.2. Adjusted odds by age and self-rated ability to complete physical daily activities of developing long COVID. E.3. Adjusted odds by age and pre-infection symptom incidences of developing long COVID. (DOCX) [file pone.0330793.s015.docx]

**Table E.1. Interaction: Age by functional status inferred from pre-infection EHR clinical observations**

| **Age (years)** | **Some prior difficulty** | **Severe prior difficulty** |
| --- | --- | --- |
| 25 | 1.06 (0.68, 1.6), 0.80 | 1.21 (0.70, 2.1), <0.49 |
| 45 | 1.03 (0.70, 1.5), 0.87 | 1.31 (0.86, 2.0), <0.21 |
| 65 | 1.01 (0.70, 1.4), 0.98 | 1.42 (0.98, 2.1), 0.07 |
| 75 | 0.98 (0.67, 1.40), 0.92 | 1.54 (1.01, 2.4), <0.04 |

Table E.1. Caption. Adjusted odds by age and pre-infection functional performance level (EHR code under "Finding of Functional Performance and Activity") of developing long COVID) compared to no prior difficulty.

**Table E.2. Interaction: Age by pre-infection self-reported physical ability.**

| **Age (years)** | **Mostly** | **A little bit** | **Not at all** |
| --- | --- | --- | --- |
| 25 | 1.06 (0.93, 1.21), 0.36 | 1.05 (0.84, 1.30), 0.68 | 1.09 (0.66, 1.78), 0.74 |
| 45 | 1.05 (0.98, 1.13), 0.18 | 0.99 (0.87, 1.12), 0.86 | 0.94 (0.71, 1.23), 0.65 |
| 65 | 1.04 (0.98, 1.11), 0.17 | 0.94 (0.85, 1.03), 0.17 | 0.81 (0.66, 0.99), 0.04 |
| 75 | 1.03 (0.94, 1.14), 0.52 | 0.88 (0.75, 1.05), 0.6 | 0.70 (0.48, 1.01), 0.06 |

Table E.2. Caption: Adjusted odds by age and self-rated ability to complete physical daily activities of developing long COVID, compared to “completely” able.

Table E.3. Interaction: Age by pre-infection total incident symptoms.

| **Age (years)** | **0 symptoms** | **1 symptom** | **7 symptoms** |
| --- | --- | --- | --- |
| 25 | 0.22 (0.05, 0.90), 0.04 | 0.47 (0.16, 1.37), 0.17 | 1.37 (0.47, 4.02), 0.56 |
| 45 | 0.18 (0.04, 0.74), 0.02 | 0.42 (0.15, 1.23), 0.11 | 1.48 (0.51, 4.31), 0.47 |
| 65 | 0.15 (0.04, 0.61), 0.01 | 0.38 (0.13, 1.11), 0.08 | 1.6 (0.55, 4.65), 0.39 |
| 85 | 0.12 (0.03, 0.50), <0.00 | 0.35 (0.12, 1.01), 0.05 | 1.78 (0.59, 5.04), 0.31 |

Table E.3. Caption. Adjusted odds by age and pre-infection symptom incidences of developing long COVID, compared to the median of four pre-infection symptoms.
