# Supplementary material for: Long COVID risk by pre-infection symptoms and functional status: A retrospective cohort study of data from the All of Us Research Program
Source: PLoS One. 2026 Jun 16;21(6):e0330793. doi: 10.1371/journal.pone.0330793 (PMC13271467; doi:10.1371/journal.pone.0330793)
Supplement: S16 Table — Correlation matrix of Pearson’s R2 correlation coefficient for all pre-infection incidences of long COVID symptoms included in the model. (DOCX) [file pone.0330793.s016.docx]

**Table E.1. Correlation matrix of pre-infection symptoms.**

|  | abdominal | anxiety | Chest pain | cough | depression | diarrhea | dizziness | dyspnea | fatigue | fever | headache | cognition | joint_pain | me_cfs | menstruation | muscle_pain | msk_chest_pain | palpitations | paraesthesia | sexual_function | rash | sleep | tachycardia |
| --- | --- | --- | --- | --- | --- | --- | --- | --- | --- | --- | --- | --- | --- | --- | --- | --- | --- | --- | --- | --- | --- | --- | --- |
| abdominal | 1.00 | 0.25 | 0.32 | 0.23 | 0.25 | 0.32 | 0.22 | 0.25 | 0.23 | 0.19 | 0.27 | 0.13 | 0.24 | 0.09 | 0.16 | 0.19 | 0.14 | 0.15 | 0.14 | 0.07 | 0.18 | 0.20 | 0.17 |
| anxiety | 0.25 | 1.00 | 0.24 | 0.18 | 0.53 | 0.20 | 0.19 | 0.19 | 0.22 | 0.12 | 0.24 | 0.22 | 0.21 | 0.09 | 0.13 | 0.16 | 0.12 | 0.14 | 0.12 | 0.07 | 0.17 | 0.29 | 0.16 |
| Chest pain | 0.32 | 0.24 | 1.00 | 0.29 | 0.23 | 0.22 | 0.27 | 0.40 | 0.24 | 0.18 | 0.28 | 0.16 | 0.28 | 0.09 | 0.05 | 0.20 | 0.33 | 0.22 | 0.16 | 0.04 | 0.16 | 0.25 | 0.21 |
| cough | 0.23 | 0.18 | 0.29 | 1.00 | 0.19 | 0.20 | 0.20 | 0.33 | 0.22 | 0.24 | 0.22 | 0.12 | 0.26 | 0.08 | 0.04 | 0.17 | 0.11 | 0.13 | 0.12 | 0.03 | 0.18 | 0.21 | 0.16 |
| depression | 0.25 | 0.53 | 0.23 | 0.19 | 1.00 | 0.20 | 0.19 | 0.20 | 0.23 | 0.12 | 0.24 | 0.26 | 0.23 | 0.10 | 0.11 | 0.16 | 0.11 | 0.10 | 0.12 | 0.06 | 0.16 | 0.31 | 0.15 |
| diarrhea | 0.32 | 0.20 | 0.22 | 0.20 | 0.20 | 1.00 | 0.20 | 0.21 | 0.21 | 0.20 | 0.21 | 0.12 | 0.18 | 0.08 | 0.06 | 0.15 | 0.11 | 0.11 | 0.11 | 0.04 | 0.16 | 0.18 | 0.17 |
| dizziness | 0.22 | 0.19 | 0.27 | 0.20 | 0.19 | 0.20 | 1.00 | 0.25 | 0.26 | 0.14 | 0.27 | 0.15 | 0.21 | 0.10 | 0.04 | 0.16 | 0.10 | 0.21 | 0.15 | 0.04 | 0.14 | 0.20 | 0.16 |
| dyspnea | 0.25 | 0.19 | 0.40 | 0.33 | 0.20 | 0.21 | 0.25 | 1.00 | 0.26 | 0.20 | 0.23 | 0.14 | 0.22 | 0.09 | 0.00 | 0.17 | 0.12 | 0.21 | 0.13 | 0.01 | 0.14 | 0.27 | 0.24 |
| fatigue | 0.23 | 0.22 | 0.24 | 0.22 | 0.23 | 0.21 | 0.26 | 0.26 | 1.00 | 0.17 | 0.22 | 0.16 | 0.24 | 0.34 | 0.07 | 0.21 | 0.12 | 0.17 | 0.17 | 0.05 | 0.18 | 0.26 | 0.14 |
| fever | 0.19 | 0.12 | 0.18 | 0.24 | 0.12 | 0.20 | 0.14 | 0.20 | 0.17 | 1.00 | 0.17 | 0.09 | 0.12 | 0.05 | 0.04 | 0.12 | 0.08 | 0.09 | 0.07 | 0.02 | 0.12 | 0.12 | 0.20 |
| headache | 0.27 | 0.24 | 0.28 | 0.22 | 0.24 | 0.21 | 0.27 | 0.23 | 0.22 | 0.17 | 1.00 | 0.17 | 0.21 | 0.08 | 0.12 | 0.21 | 0.13 | 0.15 | 0.16 | 0.06 | 0.16 | 0.20 | 0.17 |
| cognition | 0.13 | 0.22 | 0.16 | 0.12 | 0.26 | 0.12 | 0.15 | 0.14 | 0.16 | 0.09 | 0.17 | 1.00 | 0.13 | 0.07 | 0.01 | 0.10 | 0.06 | 0.06 | 0.09 | 0.02 | 0.09 | 0.17 | 0.11 |
| joint_pain | 0.24 | 0.21 | 0.28 | 0.26 | 0.23 | 0.18 | 0.21 | 0.22 | 0.24 | 0.12 | 0.21 | 0.13 | 1.00 | 0.10 | 0.04 | 0.23 | 0.15 | 0.14 | 0.17 | 0.05 | 0.19 | 0.28 | 0.11 |
| ME/CFS | 0.09 | 0.09 | 0.09 | 0.08 | 0.10 | 0.08 | 0.10 | 0.09 | 0.34 | 0.05 | 0.08 | 0.07 | 0.10 | 1.00 | 0.05 | 0.11 | 0.06 | 0.07 | 0.09 | 0.03 | 0.08 | 0.13 | 0.04 |
| menstruation | 0.16 | 0.13 | 0.05 | 0.04 | 0.11 | 0.06 | 0.04 | 0.00 | 0.07 | 0.04 | 0.12 | 0.01 | 0.04 | 0.05 | 1.00 | 0.05 | 0.04 | 0.05 | 0.05 | 0.10 | 0.11 | 0.02 | 0.06 |
| muscle_pain | 0.19 | 0.16 | 0.20 | 0.17 | 0.16 | 0.15 | 0.16 | 0.17 | 0.21 | 0.12 | 0.21 | 0.10 | 0.23 | 0.11 | 0.05 | 1.00 | 0.16 | 0.11 | 0.16 | 0.06 | 0.14 | 0.18 | 0.10 |
| msk_chest_pain | 0.14 | 0.12 | 0.33 | 0.11 | 0.11 | 0.11 | 0.10 | 0.12 | 0.12 | 0.08 | 0.13 | 0.06 | 0.15 | 0.06 | 0.04 | 0.16 | 1.00 | 0.08 | 0.10 | 0.03 | 0.10 | 0.12 | 0.08 |
| palpitations | 0.15 | 0.14 | 0.22 | 0.13 | 0.10 | 0.11 | 0.21 | 0.21 | 0.17 | 0.09 | 0.15 | 0.06 | 0.14 | 0.07 | 0.05 | 0.11 | 0.08 | 1.00 | 0.10 | 0.03 | 0.10 | 0.13 | 0.18 |
| paraesthesia | 0.14 | 0.12 | 0.16 | 0.12 | 0.12 | 0.11 | 0.15 | 0.13 | 0.17 | 0.07 | 0.16 | 0.09 | 0.17 | 0.09 | 0.05 | 0.16 | 0.10 | 0.10 | 1.00 | 0.03 | 0.11 | 0.14 | 0.07 |
| sexual_function | 0.07 | 0.07 | 0.04 | 0.03 | 0.06 | 0.04 | 0.04 | 0.01 | 0.05 | 0.02 | 0.06 | 0.02 | 0.05 | 0.03 | 0.10 | 0.06 | 0.03 | 0.03 | 0.03 | 1.00 | 0.05 | 0.04 | 0.02 |
| rash | 0.18 | 0.17 | 0.16 | 0.18 | 0.16 | 0.16 | 0.14 | 0.14 | 0.18 | 0.12 | 0.16 | 0.09 | 0.19 | 0.08 | 0.11 | 0.14 | 0.10 | 0.10 | 0.11 | 0.05 | 1.00 | 0.15 | 0.10 |
| sleep | 0.20 | 0.29 | 0.25 | 0.21 | 0.31 | 0.18 | 0.20 | 0.27 | 0.26 | 0.12 | 0.20 | 0.17 | 0.28 | 0.13 | 0.02 | 0.18 | 0.12 | 0.13 | 0.14 | 0.04 | 0.15 | 1.00 | 0.12 |
| tachycardia | 0.17 | 0.16 | 0.21 | 0.16 | 0.15 | 0.17 | 0.16 | 0.24 | 0.14 | 0.20 | 0.17 | 0.11 | 0.11 | 0.04 | 0.06 | 0.10 | 0.08 | 0.18 | 0.07 | 0.02 | 0.10 | 0.12 | 1.00 |

Table E.4 Legend: Correlation matrix of Pearson’s *R^2^* correlation coefficient for all long COVID symptoms included in the model.
